# Prolonged Culture Negativity in Infective Endocarditis Following Short-Term Antibiotic Exposure: A Case Report

**DOI:** 10.7759/cureus.99497

**Published:** 2025-12-17

**Authors:** Natsuki Mohri, Kiyofumi Ohkusu, Nobuyuki Yoshitani, Yuri Chomei, Sho Nishimura

**Affiliations:** 1 Department of Infectious Diseases, Hyogo Prefectural Harima-Himeji General Medical Center, Himeji, JPN; 2 Department of Microbiology, Tokyo Medical University, Tokyo, JPN; 3 Department of Cardiovascular Surgery, Hyogo Prefectural Harima-Himeji General Medical Center, Himeji, JPN

**Keywords:** 16s rrna pcr, blood culture-negative endocarditis, infective endocarditis, prior antibiotic exposure, streptococcus mitis

## Abstract

Blood culture-negative endocarditis remains diagnostically challenging, especially after antibiotic exposure. We report the case of a 63-year-old woman with culture-negative infective endocarditis (IE), in whom cultures were likely rendered negative due to prior antibiotic therapy consisting of levofloxacin and ceftriaxone for 12 days more than 30 days earlier and two doses of cefazolin administered 12 days before presentation. All blood and valve cultures were negative, but 16S rRNA polymerase chain reaction (PCR) identified *Streptococcus mitis* (group), guiding targeted therapy. This case illustrates that antibiotic exposure more than 10 days prior to sampling may still yield negative cultures and emphasizes the diagnostic utility of molecular tools. Clinicians should consider blood culture-negative endocarditis even after extended antibiotic-free periods.

## Introduction

Blood culture-negative endocarditis is defined as infective endocarditis (IE) in which no causative microorganism is identified by standard blood culture techniques despite appropriate incubation and subculturing [1]. This condition poses markedly diagnostic and therapeutic challenges and is associated with higher in-hospital mortality than blood culture-positive endocarditis, with reported rates of 20.1% and 16.4%, respectively [2]. A meta-analysis involving 867 patients with infective endocarditis found that 11.7% had negative blood cultures; among these, 36.1% had received antimicrobial therapy before blood sample collection, highlighting prior antibiotic exposure as a well-established risk factor for blood culture-negative endocarditis [3]. However, the optimal interval between the last antibiotic and blood culture collection to avoid false-negative results remains unclear.

In this report, we present a case of blood culture-negative endocarditis caused by *Streptococcus mitis* (group), identified through polymerase chain reaction (PCR) analysis of the excised valve tissue. Notably, the patient had not received antibiotics for 12 days prior to blood culture collection and had completed an earlier antibiotic course more than 30 days beforehand; however, the cultures remained negative even after 21 days of extended incubation. This reinforces the notion that even remote antibiotic exposure can suppress bacteremia to undetectable levels and highlights the diagnostic value of molecular testing in suspected blood culture-negative endocarditis.

## Case presentation

A 63-year-old woman with no significant medical history, including no known structural heart disease, presented with a six-week history of generalized malaise and persistent fever exceeding 38°C for approximately one month. The patient was initially evaluated at a community clinic and treated empirically with a two-day course of intravenous ceftriaxone (initiated 42 days before the second hospitalization, during which infective endocarditis (IE) was diagnosed), followed by a 10-day course of oral levofloxacin (250 mg daily). This resulted in partial clinical improvement, with her temperature decreasing to approximately 37°C, but a low-grade fever in the 37°C range and mild inflammation (C-reactive protein (CRP): 1-2 mg/dL) persisted. Eighteen days later (12 days before the second admission), the patient underwent elective surgery for an obturator hernia. On the day of surgery, the patient received two prophylactic doses of intravenous cefazolin. The patient recovered well during the seven-day postoperative hospital stay. The patient was discharged without any complications.

Six days after discharge, the patient suddenly developed back pain and bilateral leg numbness. Contrast-enhanced CT revealed occlusion of the superior mesenteric artery and occlusion extending from the terminal aorta to the bilateral iliac arteries, resulting in a second admission. On hospital day 1, percutaneous transluminal angioplasty with endovascular stent placement was performed from the distal aorta to the right common iliac artery, from the right common iliac artery to the right external iliac artery, and in the celiac artery, during which cefazolin was administered as a perioperative antibiotic and continued postoperatively. Two sets of blood cultures collected on the day of admission, prior to the perioperative administration of cefazolin, yielded negative results.

**Figure 1:**
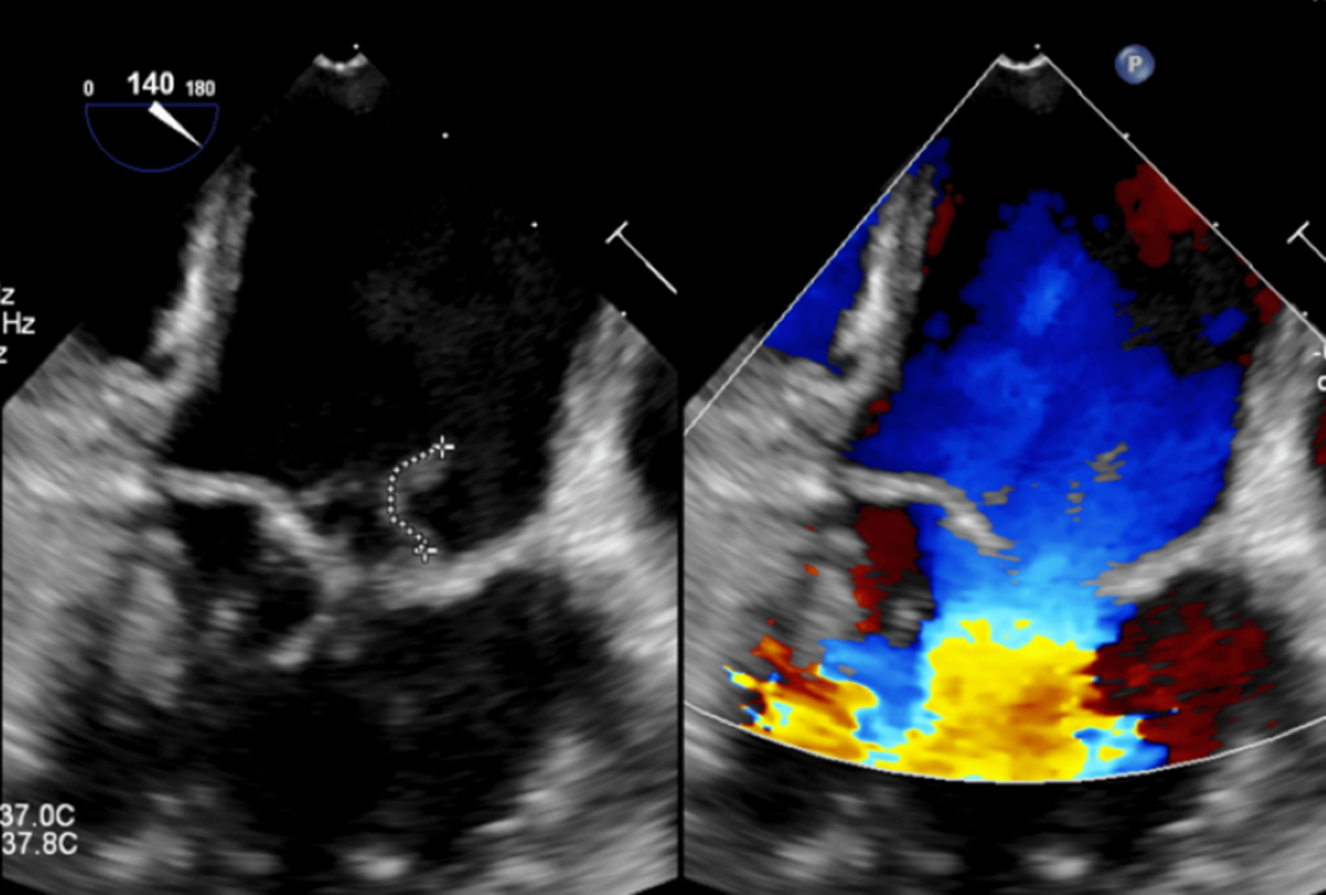
Transesophageal echocardiography showing mitral valve vegetations Transesophageal echocardiography revealed new-onset severe mitral regurgitation that was not observed on preoperative imaging. Two mobile, filamentous echo densities measuring 7.4 mm and 11.6 mm were visualized on the A2 segment of the anterior mitral valve leaflet, suggestive of vegetations.

At admission, preoperative transthoracic echocardiography showed only mild mitral regurgitation without vegetations. On hospital day 7, a predischarge follow-up transthoracic echocardiography revealed new-onset severe mitral regurgitation incidentally, which was not detected during preoperative echocardiography performed on the day of admission. Furthermore, two mobile filamentous echo densities attached to the A2 segment of the anterior mitral valve leaflet were observed, indicating vegetation. Transesophageal echocardiography identified mobile vegetations measuring 7.4 mm and 11.6 mm arising from the lateral aspect of the A2 segment (Figure 1). The patient met the 2023 Duke-International Society for Cardiovascular Infectious Diseases (Duke-ISCVID) criteria for possible infective endocarditis, fulfilling one major criterion (positive echocardiographic findings) and two minor criteria (fever and vascular phenomena) [4]. Laboratory findings during the first and second hospitalizations, as well as on hospital day 7 when infective endocarditis was diagnosed, are summarized in Table 1.

**Table 1:** Laboratory findings at the first hospitalization, the second hospitalization, and on hospital day 7 when infective endocarditis was diagnosed

Laboratory parameter (unit)	Reference range	1st hospitalization	2nd hospitalization	Diagnosis of infective endocarditis (hospital day 7)
White blood cell count (10^3^/μL)	3.3-8.6	13.0	20.6	11.6
Hemoglobin (g/dL)	11.6-14.8	10.9	10.9	10.8
Platelet count (10^3^/μL)	158-348	427	356	473
C-reactive protein (mg/dL)	0.00-0.14	1.53	5.95	2.50

**Figure 2:**
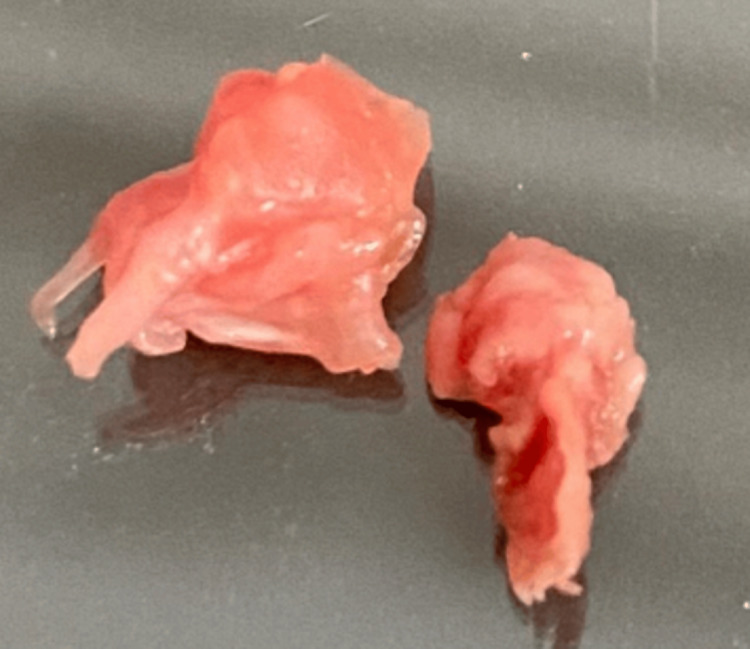
Gross appearance of the resected anterior mitral valve segments The specimen on the left shows the A2 segment of the anterior mitral leaflet with attached chordae tendineae. The specimen on the right corresponds to the A1 segment. Both were surgically excised because of infective endocarditis.

**Figure 3:**
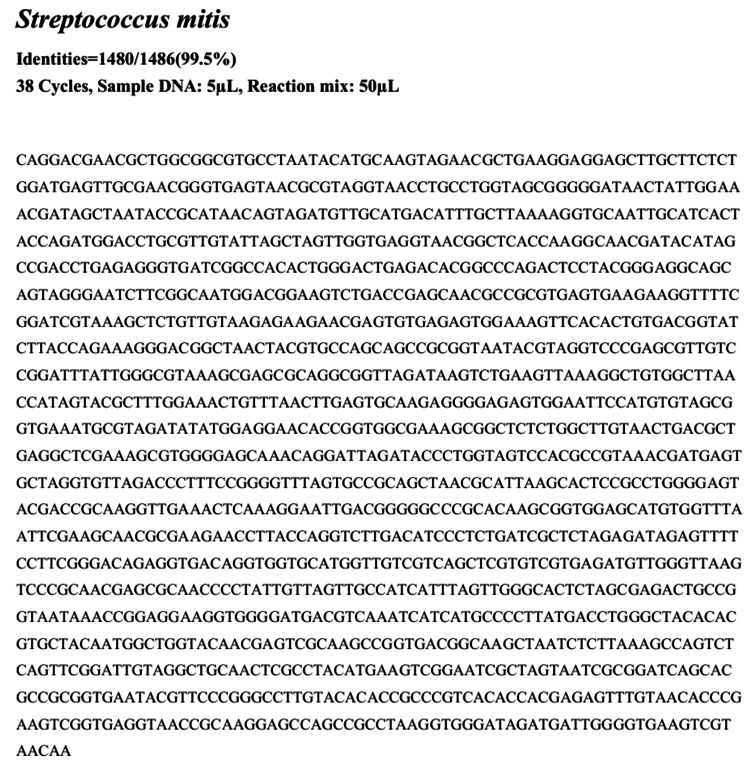
PCR amplification and sequencing results identifying from the resected tissue 16S rRNA gene PCR was performed to detect bacterial DNA from the resected tissue. Subsequent sequencing of the amplified product revealed a 99.5% identity (1480/1486 bp) within the *Streptococcus mitis* (group), confirming the presence of this organism in the specimen. PCR: polymerase chain reaction

On hospital day 8, the patient underwent mitral valve plasty with vegetation resection because a mobile vegetation >10 mm was present on the A1-A2 segments of the anterior leaflet, and valve tissue evaluation was warranted given persistently negative blood cultures; the resected area included a small perforation. The resected mitral valve tissue and chordae tendineae are shown in Figure 2. A smear of the vegetation did not reveal bacterial organisms, but histopathologic examination of the excised valve tissue demonstrated neutrophilic infiltration with no thrombus and no malignant cells. Two sets of preoperative blood cultures (collected on hospital days 0 and 7) and intraoperative valve cultures remained negative after 21 days of extended incubation. Given histopathologic findings consistent with infective endocarditis and the concern for culture-negative disease in the context of prior antibiotic exposure, we performed broad-range 16S rRNA PCR on excised valve tissue using universal primers 27F and 1492R [5,6]. The amplicon was Sanger-sequenced and queried with NCBI BLAST and EzTaxon (EzBioCloud). The sequence showed 99.5% (1480/1486 bp) identity within the *Streptococcus mitis* (group) (Figure 3). The possibility of *S. pneumoniae* was excluded because the lytA gene was negative by real-time PCR testing. The primers and methods used for the lytA gene detection were based on Fukasawa et al. [7].

**Figure 4:**
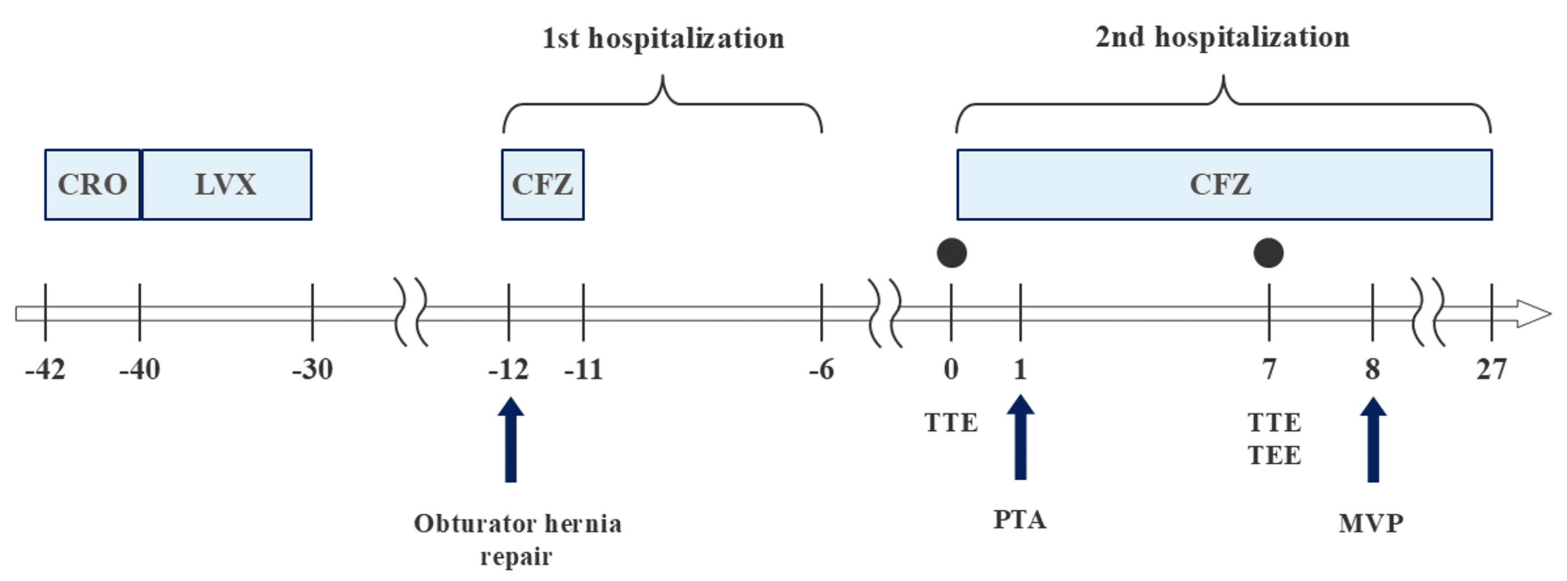
Clinical timeline showing the patient's treatment course, hospitalizations, and key interventions This timeline illustrates the chronological sequence of antimicrobial therapy, surgical procedures, and clinical events. The black circle (●) indicates the timing of blood culture collection. CFZ: cefazolin, CRO: ceftriaxone, LVX: levofloxacin, MVP: mitral valve plasty, PTA: percutaneous transluminal angioplasty, TEE: transesophageal echocardiography, TTE: transthoracic echocardiography

The patient received intravenous cefazolin for four weeks after valve surgery for infective endocarditis caused by *S. mitis* (group). At the time of surgery, pathogen identification was pending; we selected cefazolin as a narrow-spectrum empiric agent while awaiting molecular results, with a contingency to repeat cultures if PCR was infeasible or non-diagnostic. After 16S rRNA sequencing identified the *S. mitis* group, and in light of our institutional antibiogram showing good beta-lactam susceptibility, cefazolin was continued. Given the possibility that the arterial occlusions treated with stent placement represented septic embolic events, the patient was administered oral amoxicillin for a planned duration of approximately six months. Amoxicillin was selected based on our institutional antibiogram that shows that *Streptococcus *species isolated from IE cases at our facility exhibit very high susceptibility to penicillin. Postoperatively, no new embolic events occurred, and follow-up echocardiography showed improvement of mitral regurgitation to trace-mild. A summary of the patient's clinical course and antimicrobial therapy is shown in Figure 4.

## Discussion

Prior antibiotic administration is a major contributor to negative blood cultures in patients with endocarditis. Studies have shown that 35%-74% of blood culture-negative endocarditis cases involve recent antibiotic use [8]. In a prospective intensive care unit (ICU) cohort at sepsis onset, Scheer et al. found that blood culture positivity was 50.6% (78/154) when cultures were drawn before antibiotics versus 27.7% (112/405) during antibiotic therapy; antibiotic use remained an independent predictor of reduced positivity (adjusted odds ratio (OR): 0.4, 95% confidence interval (CI): 0.3-0.6). In a paired subgroup of 35 patients who had cultures both before and after antibiotic initiation, positivity declined from 57.1% (20/35) to 25.7% (9/35) (p=0.008) [9]. Our case highlights that both a brief inpatient exposure 12 days before sampling and an earlier 12-day course completed more than 30 days previously could have contributed to culture negativity; while the earlier, longer course is likely the stronger driver, the absence of interim blood cultures precludes determining their relative impact. These findings highlight the need to consider the impact of prior antibiotic exposure, even after a seemingly adequate antibiotic-free interval, when evaluating suspected blood culture-negative endocarditis.

Additionally, the patient received a 12-day course of antibiotic therapy with ceftriaxone, followed by oral levofloxacin for more than 30 days prior to presentation, which may have contributed to culture negativity and led to partially treated infective endocarditis.

Molecular diagnostic techniques are essential tools for identifying the causative organisms of blood culture-negative endocarditis when blood cultures fail. In our case, broad-range PCR of the excised valve tissue identified *Streptococcus mitis*, thereby guiding the appropriate antimicrobial therapy. Harris et al. demonstrated that 16S rRNA PCR could detect pathogens in 62% of culture-negative endocarditis cases, including viridans group streptococci [10]. This supports the diagnostic value of PCR in our case. Halavaara et al. reported, in a cohort of 87 surgical IE cases, that with <14 days of preoperative antibiotics, valve culture was positive in 41% (19/46), whereas 16S rRNA PCR was positive in 91% (42/46); with ≥14 days, all valve cultures were negative (0/34), while PCR remained positive in 53% (18/34). PCR identified a pathogen in 77% (10/13) of blood culture-negative IE. These data underscore the rapid loss of culture yield after only days of therapy, contrasted with the more stable performance of 16S rRNA PCR when culture sensitivity is compromised [11]. These findings support the utility of molecular diagnostics for resolving cases where traditional culture-based methods are inadequate.

## Conclusions

The duration of the antibiotic-free interval required to restore blood culture sensitivity in patients with suspected endocarditis remains unknown. This case suggests that even a single dose of antibiotics administered approximately two weeks prior or a 12-day course of antibiotics given one month earlier may have played a role in rendering negative blood cultures in the context of infective endocarditis. These findings emphasize that even short and inadequate courses of antibiotics administered long before presentation should be considered when assessing patients with blood culture-negative endocarditis. Importantly, this case highlights the diagnostic value of broad-range 16S rRNA PCR in identifying causative organisms when conventional culture fails. Molecular diagnostics should be incorporated early into the diagnostic workup for blood culture-negative endocarditis, particularly in patients with a history of antimicrobial therapy.
